# Detection of Migrainous Infarction with Formal Visual Field Testing: A Case Report

**DOI:** 10.5811/cpcem.2020.4.46387

**Published:** 2020-07-09

**Authors:** William Bylund, Ross Patrick, Ann Macdonald

**Affiliations:** * Naval Hospital Okinawa, Department of Emergency Medicine, Okinawa, Japan; † Naval Hospital Okinawa, Department of Internal Medicine, Okinawa, Japan; ‡ Naval Hospital Okinawa, Department of Optometry, Okinawa, Japan

**Keywords:** cerebrovascular accident, homonymous hemianopsia, migraine

## Abstract

**Introduction:**

Cerebrovascular accidents (CVA) of the posterior circulation are a rare complication of migraine, and present with atypical CVA symptomatology.

**Case Report:**

A 49-year-old-male presented with complaint of persistent visual aura and resolved mild cephalgia. His exam corroborated his reported incomplete left inferior quadrantanopia, and was confirmed by immediate formal optometry evaluation. Occipital CVA was confirmed on admission.

**Conclusion:**

Migrainous strokes of posterior circulation should be considered as a potential diagnosis in any headache patient with persistent visual aura. This case suggests that incorporation of formal visual field testing in the emergent setting can shorten the time required for diagnosis in certain circumstances.

## INTRODUCTION

Posterior circulation ischemic stroke syndromes comprise approximately 20% of stroke syndromes, and often present with atypical symptoms when compared to anterior or middle circulation ischemic strokes.^1^ However, most strokes do affect the visual pathway, some causing oculomotor deficits, while up to 70% result in decreased visual acuity.^2^ This high percentage is not surprising when one considers the multiple anatomic regions involved in visual processing, visuospatial reasoning, and oculomotor control. Information from the retina travels through the optic nerves, optic chiasm, optic tracts, lateral geniculate nuclei, and the optic radiations, before finally arriving to the visual cortex in the occipital lobes. Each of these structures is vulnerable to ischemia with variable clinical effects. Although the posterior circulation only supplies 20% of the brain, it is critical to consciousness, movement, and visual processing in the occipital cortex.^1^, ^3^

Acute ischemic strokes are considered an essential diagnosis to make quickly in the emergency department (ED), in part because of their significant morbidity and mortality. There are nearly 800,000 strokes annually in the United States, and approximately 17% result in death.^3^–^4^ Given the magnitude of the problem, the American Heart Association has established multidisciplinary programs to improve outcomes.^5^ In the 1990s, the National Institutes of Health Stroke Scale (NIHSS) was developed to better assess cerebrovascular accident (CVA) severity, although the NIHSS does not well assess posterior circulation strokes.^1^ Migrainous infarctions are a rare complication of migraines, representing only 0.2–0.5% of all ischemic strokes.^6^ According to the International Classification of Headache Disorders, migrainous infarction is defined as “One or more migraine aura symptoms occurring in association with an ischemic brain lesion in the appropriate territory demonstrated by neuroimaging, with onset during the course of a typical migraine with aura attack.”^7^ The following case demonstrates an unusual presentation of CVA that qualifies as a migrainous infarction. Visual field images demonstrate the diagnostic value of objective optometry evaluation.

## CASE REPORT

**Image 1:**
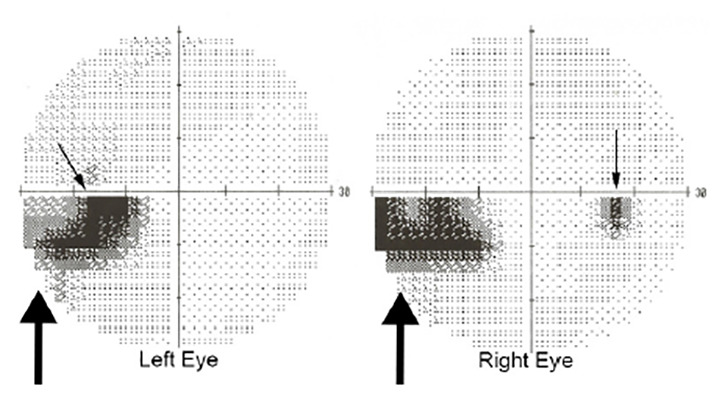
Visual field tests via Humphrey visual field 24-2 Swedish Interactive Threshold Algorithm Fast algorithm: At left, the large arrow points to a clustered inferior temporal visual field defect adjacent and inferior to the anatomic blind spot (small arrow). At right, the large arrow points to a nasal inferior defect, while the clustered inferior temporal points correlate the anatomic blind spot (small arrow). Together, these images demonstrate a homonymous defect secondary to infarct.

A 49-year-old male with relevant medical history of migraines and hyperlipidemia (for which he was on 20 milligram [mg] daily atorvastatin) presented to our hospital after approximately 12 hours of decreased vision in his left lower visual field. Patient’s history was notable for this quadrantanopia being consistent with prior migraines; however, he decided to present to the ED as this presentation was significantly different from his usual migraine duration. The headache had nearly resolved on presentation to the ED, but the patient still complained of decreased vision. The majority of the patient’s exam was normal, including vital signs, cranial nerves II–XII, coordination, gait and balance. Confrontational visual fields were concerning for possible incomplete left inferior homonymous quadrantanopia. His electrocardiogram demonstrated normal sinus rhythm with no ischemic changes. Non-contrast computed tomography (CT) of the head was obtained and revealed no bleeding or mass. Magnetic resonance imaging (MRI) of the brain was ordered, but was not immediately available. Optometry service was consulted to confirm visual field defects and performed the Humphrey visual field 24-2 Swedish Interactive Threshold Algorithm Fast, which tests 54 visual field data points per eye and takes approximately five minutes to perform depending on the reliability of the test and size of the defect. Test reliability aids the provider in assigning diagnostic value. The optometrist’s exam was reliable and consistent with left inferior incomplete homonymous quadrantanopia (Image 1). The patient was administered 324 mg of chewable aspirin but given his delayed presentation and low NIHSS, systemic thrombolytics were not administered. After consultation with internal medicine, the patient was admitted to a telemetry-capable ward for further evaluation and management.

**CPC-EM Capsule**

What do we already know about this clinical entity?

Migraines are associated with cerebrovascular accidents (CVA); isolated quadrantopia leading to a stroke diagnosis has only been described once previously.

What makes this presentation of disease reportable?

This is the second case of migrainous infarction presenting with isolated visual field deficit and highlights the utility of formal visual field testing.

What is the major learning point?

Physicians should consider CVA in the setting of migraine, and use formal visual field testing to delineate a suspected homonymous defect when magnetic resonance imaging is unavailable.

How might this improve emergency medicine practice?

Improved recognition of acute CVA presentation and potential diagnostic modalities can improve timely diagnosis, enabling earlier treatment in certain instances.

**Image 2:**
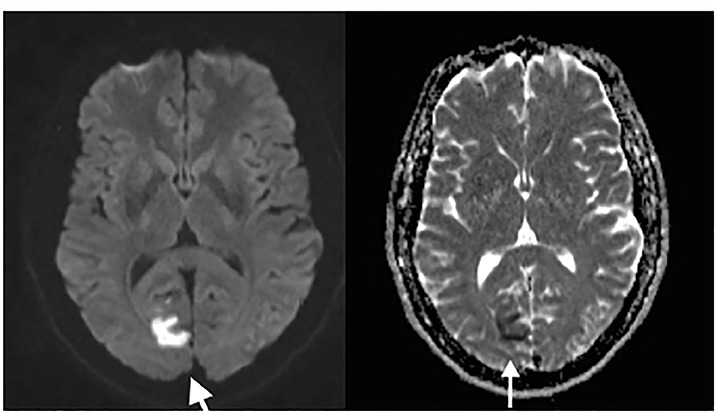
Transverse brain magnetic resonance imaging at level of the basal ganglia: At left, an axial diffusion weighted image demonstrates hyperintense signal of right mesial occipital lobe. At right, the apparent diffusion coefficient illustrates corresponding hypointensity, which is consistent with acute brain ischemia.

A brain MRI with angiography was obtained the following day, which demonstrated acute cerebral infarction within the medial aspect of the right occipital lobe and small infarcts within the right cerebellum and the vermis (Image 2). There was also 50% luminal narrowing at the origin of the left vertebral artery, and scattered narrowing of less than 50% in the bilateral internal carotid arteries.

The following day, the patient reported no change in his visual symptoms. Neurology recommended increase of his aspirin to 325 mg daily as well as continuation of atorvastatin increased to 80 mg daily. Transthoracic echocardiogram was performed, and a two-day Holter (followed by 12-day cardiac event monitor) was initiated. The patient was discharged on ischemic secondary prevention therapy as noted above, pending cardioembolic etiology rule out.

Implanted loop recorder (ILR) was arranged by referral to a capable tertiary center in Okinawa, Japan. Transthoracic echocardiogram with bubble study interpretation resulted days later, revealing early positive agitated saline study consistent with patent foramen ovale (PFO). Additionally, neurology started a hypercoagulability workup to include assessment of lupus anticoagulant, protein C/S panel, factor V Leiden mutation, antithrombin III activity, and prothrombin mutation. Only the prothrombin workup was abnormal, revealing a heterozygous mutation (G-20210-A). However, in the absence of venous thromboembolism, prolonged antithrombotic therapy is generally not recommended for prothrombin G20210A heterozygotes.^8^

At the tertiary center, the patient underwent ILR placement, and a transesophageal echocardiogram was performed. The latter ruled out intracardiac thrombus, and demonstrated right to left shunting via his PFO. However, cardiology stated PFO closure was not indicated due to cryptogenic stroke etiology with unfavorable risk-benefit analysis. The ILR showed no cardiac dysrhythmias. In the first month after discharge, the patient had mild improvement in vision subjectively. At three-week ophthalmology follow-up, he had similar improvement on repeat visual field testing, and was cleared to return to driving. In an eight-month follow-up telephone consult, the patient stated he had no further improvement in vision, and had no recurrent strokes despite increased awareness of stroke symptoms. Repeat visual field testing performed at three and eight months showed no significant improvement or worsening.

## DISCUSSION

This case highlights the importance of thorough visual field exam in diagnosing posterior CVA, which may have significant ramifications on immediate treatment. Additionally, this case meets criteria for migrainous infarction, although the exact pathophysiology of this case cannot be definitively proven and the etiology of migrainous infarctions at large remains debated.^9^–^10^ Symptoms of posterior circulation strokes can be subtle, go unnoticed by the patient, and result in delayed diagnosis.^1^,^11^ Our patient’s presentation was delayed because he did not recognize his symptoms were consistent with a stroke, only becoming suspicious that something was amiss when his usual symptoms failed to resolve. While homonymous hemianopia is well reported,^13^–^14^ to our knowledge, there is only one similar case of isolated homonymous quadrantanopia in the emergent setting that ultimately led to a diagnosis of CVA. Even then, the diagnosis was only made following an outpatient neuro-ophthalmology workup, leading the authors to recommend increased utilization of objective visual field tests.^15^

We found four ophthalmology case series related to isolated quadrantanopia or homonymous hemianopsia, including a combined total of 1050 patients. The studies show that approximately 80% of homonymous visual field defects are due to ischemic strokes, most often of the occipital lobe, in elderly patients, and without associated neurological findings. These case series are substantially different than our case report because the exam findings were made in a delayed setting by an eye specialist.^11^–^14^ Based on the composition and number of reported cases, it is possible a significant number of isolated posterior strokes may be missed on initial presentation. The fact that no literature could be located on emergent formal visual field assessment may suggest less diagnostic utility in an era when rapid MRI acquisition is common. However, remote locations may occasionally find value in this less expensive, functional test.

Our case report and existing literature illustrate the diagnostic challenge of certain stroke subtypes and the utility of formal visual field testing by an eye specialist when there is suspicion of a posterior cerebral vascular event. The homonymous defect could have been missed, as it represented only 10% of the visual field. Small defects like these may explain the great variation in stroke patients with visual field loss (45–92%).^2^ This wide range may also be secondary to the low sensitivity of confrontational visual fields and the difficulty in obtaining a more thorough visual field exam in a rapid manner. In many cases, the patient may not be stable to leave the ED for a detailed visual field evaluation, or doing so would delay care beyond the standard stroke treatment window. To further obfuscate matters, the current clinical scoring standard, the NIHSS, generally scores posterior circulation strokes lower than classic stroke presentations, as dizziness and visual fields generate minimal points in comparison to motor and language deficits.^1^

Although posterior strokes and migrainous infarctions remain rare, literature suggests several associations. Patients with chronic migraines actually have twice the risk of stroke and other cardiovascular complications.^10^ The greater the frequency of migraine, the greater the risk of stroke.^10^ Consequently, some have suggested that migraine pathophysiology itself could be causal.^10^ Others have suggested that common risk factors could be the culprit.^10^ Hypothesized mechanisms for this relationship include genetic associations, endothelial dysfunction, and defects in coagulation factors.^10^ While it is possible that our patient’s heterozygous prothrombin mutation and PFO created risk factors for paradoxical embolic phenomenon, it is far more likely that the patient had migrainous vasospasm in the setting of pre-existing atherosclerotic disease, given the odds of a paradoxical emboli localizing to the same cerebral territory affected by his usual migraines is essentially nil. A PFO traditionally had been thought to predispose to migrainous infarction because the prevalence of PFO is twice as common in patients with migraines with aura.^10^ Interestingly, a randomized clinical trial of PFO closure found no effect on migraine symptoms.^16^

The treatment and prognosis for migraines and strokes generally remain two separate pathways. There are no additional recommendations to give antithrombotics to patients with chronic migraines. Calcium channel blockers and beta blockers have been used successfully for migraine prophylaxis, and further studies could possibly show a reduced cardiovascular risk in select migraine patients on these medications. Unfortunately, our patient met indications for few medications at time of presentation. His headache had resolved and his blood pressure was within normal limits. Aspirin was given, but he was not a candidate for thrombolytics based on the minor neurological deficit and the duration of symptoms. Patients with this presentation have variable prognosis. Some studies state that 44% of patients make complete visual recovery, usually within the first three months and unlikely after six months.^17^ Our patient reported similar recovery, with some recovery in the first month before a plateau in visual improvement.

## CONCLUSION

Our case demonstrates isolated homonymous quadrantanopia as an uncommon presentation of a posterior stroke, which also met criteria for migrainous infarct. There is scant emergency medicine literature related to our case; more common is ophthalmologic literature discovering homonymous defect in delayed manner.^11^,^13^ The possibility of migraine precipitating infarction remains a viable mechanism in this case. Further studies are needed to understand this relationship. Indeed, emergency physicians must consider acute ischemic cerebrovascular event when a patient presents with visual complaints. Physicians must carefully examine for any potential homonymous deficit, as subtle stroke presentations such as this may be difficult to diagnose. When the exam is not definitive and advanced imaging is unavailable, rapid formal visual field testing is a useful adjunct as rapid diagnosis may enable expedited treatment and ultimately improve outcomes.
